# Solid-State NMR Studies of the Succinate-Acetate Permease from Citrobacter Koseri in Liposomes and Native Nanodiscs

**DOI:** 10.3390/life11090908

**Published:** 2021-08-31

**Authors:** Xing-Qi Dong, Jing-Yu Lin, Peng-Fei Wang, Yi Li, Jian Wang, Bing Li, Jun Liao, Jun-Xia Lu

**Affiliations:** 1 School of Life Science and Technology, ShanghaiTech University, Shanghai 201210, China; dongxq@shanghaitech.edu.cn (X.-Q.D.); linjy@shanghaitech.edu.cn (J.-Y.L.); wangpf@shanghaitech.edu.cn (P.-F.W.); liyi@shanghaitech.edu.cn (Y.L.); wangjian1@shanghaitech.edu.cn (J.W.); libing@shanghaitech.edu.cn (B.L.); liaojun@shanghaitech.edu.cn (J.L.); 2 State Key Laboratory of Molecular Biology, CAS Center for Excellence in Molecular Cell Science, Shanghai Institute of Biochemistry and Cell Biology, Chinese Academy of Sciences, Shanghai 200031, China; 3 University of Chinese Academy of Sciences, Beijing 100049, China

**Keywords:** membrane protein complex, functional state, native membrane environment, ^1^H-detected solid-state NMR

## Abstract

The succinate-acetate permease (SatP) is an anion channel with six transmembrane domains. It forms different oligomers, especially hexamers in the detergent as well as in the membrane. Solid-state NMR studies of SatP were carried out successfully on SatP complexes by reconstructing the protein into liposomes or retaining the protein in the native membrane of *E. coli.*, where it was expressed. The comparison of ^13^C-^13^C 2D correlation spectra between the two samples showed great similarity, opening the possibility to further study the acetate transport mechanism of SatP in its native membrane environment. Solid-state NMR studies also revealed small chemical shift differences of SatP in the two different membrane systems, indicating the importance of the lipid environment in determining the membrane protein structures and dynamics. Combining different 2D SSNMR spectra, chemical shift assignments were made on some sites, consistent with the helical structures in the transmembrane domains. In the end, we pointed out the limitation in the sensitivity for membrane proteins with such a size, and also indicated possible ways to overcome it.

## 1. Introduction

A protein is a delicate and sophisticated molecular machine that is always in motion. The advantage of NMR in protein structural studies is its ability to study a protein in its action states. However, the structural characterization of membrane proteins has been difficult in that it requires a particular membrane-mimicking environment. Solution NMR studies have indicated the distortion of protein structure may occur using the detergent micelles to solubilize membrane proteins [1,2,3]. Solid-state NMR (SSNMR) studies membrane proteins in a lipid bilayer environment, such as liposomes [4,5] or nanodiscs surrounded by the scaffold proteins [6]. However, it is also very challenging to reconstitute the protein into the bilayer environment. In the process of the reconstitution of membrane proteins in the bilayer, detergent is usually required to co-solubilize the protein and lipids and is dialyzed out afterwards. It usually takes quite a long time to optimize the whole process. 

The direct study of the membrane protein in its native environment would be a good alternative. It avoids the exposure of the membrane protein to a detergent medium, thus reducing the possibility of misfolding. Zhao et al. has studied the gating mechanism of aquaporin Z in its native *E. coli* membranes [7]. The membrane components with expressed proteins were isolated using differential centrifugation without further purification. Another approach is to use the styrene maleic acid (SMA) co-polymer, which is able to strip the membrane protein off from the membrane and forms the bilayered native nanodiscs [8,9]. Native nanodiscs retain a portion of the protein’s native membrane environment and are reported to preserve the function and stability of the membrane proteins [10]. Furthermore, both sides of the bilayers are exposed to the buffered medium for the nanodiscs, different from the liposome, facilitating the ligand titration studies. The bilayered nanodisc is also different from the bilayer in the liposome in that it has a low curvature. In this paper, we explored the preparation method of liposomes and native nanodiscs for a magic-angle-spinning (MAS) SSNMR study of SatP from *Citrobacter Koseri*. 

**Figure 1:**
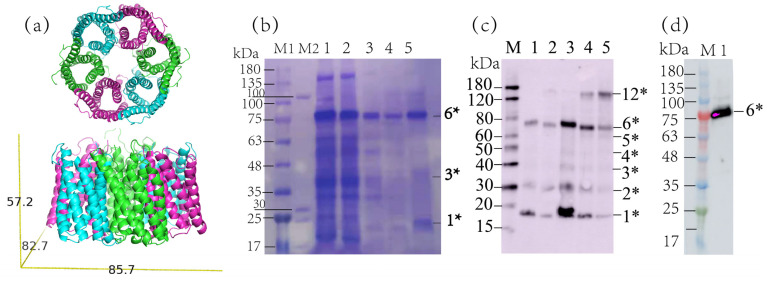
SatP protein purification and native nanodisc preparation. (**a**) The crystal structure of hexameric SatP with dimensions labeled in Å. The image was reconstructed from pdb: 5ys3 using pyMOL (Schrödinger, Inc.). (**b**) SDS-PAGE gel showed the purification results of SatP. Lane M1 and M2 are two different molecular marker lanes. The protein extraction from cell lysis was solubilized in 40 mM DM, lane 1; the flow through from Ni column, lane 2; the column wash using 0 mM imidazole, lane 3; elution using 30 mM imidazole, lane 4; elution using 300 mM imidazole, lane 5. The detergent concentration in the column wash and elution buffer was 4 mM DM. (**c**) Glutaraldehyde crosslinking of SatP in DM showed SatP in different oligomeric states. The western blot result using mouse anti-his antibody (abcam) was shown. The 0.1 mg/mL SatP, lane 1; 0.1 mg/mL SatP with 0.1% glutaraldehyde crosslinking for 5 min, lane 2; the 0.3 mg/mL SatP, lane 3; 0.3 mg/mL SatP with 0.1% glutaraldehyde crosslinking for 5 min, lane 4; 0.5 mg/mL SatP with 0.1% glutaraldehyde crosslinking for 30 min, lane 5. (**d**) Western blot showed the results of native nanodisc preparation, where lane 1 was obtained from 300 mM imidazole elution. n* in (**b**–**d**) indicates different oligomeric states of SatP.

SatP is an anion channel with six transmembrane helices that unidirectionally translocates acetate at rates in the order of ~10^7^ ions/s [11]. Acetate is an important intermediate that is required in the formation of acetyl coenzyme A, acetylation of proteins and is involved in many other signaling mechanisms [12,13,14]. Although the crystal structure of SatP is known, much is still to be understood on its acetate translocation mechanism [15]. Therefore, SSNMR would provide a unique view of SatP structure and dynamics at the atomic level in acetate translocation in a bilayer membrane environment. Based on its crystal structural studies, SatP forms hexamers (Figure 1a) [11,16]. However, it is still to be confirmed if SatP also forms hexamers in the membrane. With its monomer molecular weight of about 21 kDa, the hexameric complex would be 126 kDa, making it a very big system for NMR study.

## 2. Materials and Methods

### 2.1. Protein Expression

The full-length gene of SatP from *Citrobacter koseri* ATCC BAA-895 followed by a thrombin cleavage sequence (LVPRGS) and a (His)_6_ tag was cloned into a pQE60 vector [11]. *E. Coli* M15 cells containing the recombinant plasmid were cultured for protein expression. For ^13^C, ^15^N uniformly labeled SatP protein, the cells were cultured at 37 °C in M9 medium to OD600 ~1–1.2, and then transferred to the same volume of fresh M9 medium containing ^13^C labeled glucose (2 g/L) and ^15^N labeled ammonium chloride (1.5 g/L) [17]. The protein expression was induced after 30 min incubation of the cells in the fresh medium at 37 °C. After that, IPTG was added to a final concentration of 0.4 mM and the temperature was reduced to 18 °C for overnight expression.

### 2.2. Protein Purification and Liposome Preparation

The cells were lysed by a high-pressure homogenizer (FB-110X, Shanghai Litu Ins., China) in lysis buffer (50 mM HEPES, pH 7.3, 100 mM NaAc, 50 mM NaCl, 5 mM MgCl_2_, 0.2 mM TCEP, 1 mM PMSF) plus an additional 0.2 mM EDTA 10 μL DNase (per 50 mL of solution). The cell debris was removed by centrifuging at 9000× *g* for 15 min at 4 °C. The total cell membranes were then collected by high-speed centrifugation at 100,000× *g* for 1 h and solubilized with lysis buffer containing an additional 10 μL DNase (per 50 mL of solution) and 40 mM Decyl-β-D-maltopyranoside (DM, Anatrace, Maumee, OH, USA), at 4 °C for 3 h. Another centrifugation at 21,000× *g* for 1.5 h was applied, and the supernatant was loaded onto a Ni^2+^ affinity column (Smart-Life Science, Bandra East Mumbai, India) pre-equilibrated with solution containing 20 mM HEPES (pH 7.3), 100 mM NaAc, 50 mM NaCl, 0.2 mM TCEP and 4 mM DM. The protein was eluted with the equilibrium solution with an additional 0, 30 or 300 mM imidazole, respectively. Finally, the protein solution from 30 and 300 mM imidazole elution was kept for liposome construction. The protein solution was concentrated to a final concentration of 100 mg/mL, and imidazole was removed by buffer exchange several times using an Amicon Ultra protein concentrator (UFC910024 15 ml 30K 24pk, Millipore, Merck, KGaA, Darmstadt, Germany. 

The lipid mixture (DOPC/DOPG = 3/1 mole ratio) (Avanti Polar Lipids, Alabaster, AL, USA) was solubilized in the above equilibrium solution to a total concentration of 60 mg/mL and mixed with 100 mg/mL SatP in equal volumes to make the sample containing 30 mg/mL lipid mixture and 50 mg/mL SatP. The mixture was incubated at 37 °C for 18 h using a sample mixer setting at 40 rpm speed. Biobeads SM-2 (Bio-Rad, Hercules, California, USA) were added to about 25 times the weight of DM, and the mixture was incubated continuously at 37 °C for another 3 h. The addition of biobeads was repeated two more times. Finally, the solution was kept in the standing position to let the biobeads SM-2 settle to the bottom of the tube. The liposome formed in the solution was then transferred and the buffer was exchanged to water using an Amicon Ultra protein concentrator (UFC910024 15ml 30K, Millipore, Merck, KGaA, Darmstadt, Germany). 

### 2.3. The Preparation of Hydrolyzed SMA

Following the instruction described by Lee [18], 25 g SMA30010 (S:MA = 2.3:1) anhydride (Polyscope Polymers BV, CZ Geleen, The Netherlands) was refluxed with 250 mL 1 M NaOH at 100 °C for 6 h, until the majority of SMA was dissolved in solution. After the solution was cooled down to room temperature, excessive HCl was added until the pH was lowered to 5 (measured by pH test paper) and SMA precipitation was formed. The pellet was collected and then washed three times by resuspending it with water followed by centrifugation at 11,000× *g* for 15 min. After that, the above process was repeated by adding 0.6 M NaOH to the solution until the polymer was dissolved. The pH of the solution was adjusted to acidic conditions (pH < 5) to precipitate SMA and the wash processes were also carried out in the same way. Finally, 0.6 M NaOH was added to SMA precipitation and the mixture was vortexed until no precipitation can be found. The pH was checked and adjusted carefully to 7–8. Then the polymer solution was lyophilized to a dry powder, which was the hydrolyzed SMA. The SMA polymer solution was corrosive, so the polypropylene tube was used in the centrifugation of SMA.

### 2.4. C, ^15^N Labeled Native Nanodisc Sample Preparation

The harvested cells were lysed by a high-pressure homogenizer in the lysis buffer as described above. The cell debris was removed by centrifuging at 11,000× *g* for 15 min at 4 °C and the total cell membranes were collected by high-speed centrifugation at 100,000× *g* for 1 h at 4 °C. Then, the cell membrane was pushed through a dounce tissue grinder (Wheaton, Milville, NJ, USA) about 150 times to obtain a homogeneous suspension. After that, the suspension was centrifuged at 100,000× *g* for 1 h at 4 °C again and homogenized two more times. 

After the final centrifugation, we weighed the wet weight of the cell membrane and added buffer (40 mM Tris, 300 mM NaCl, 2 mM TCEP, pH = 7.3) to resuspend cell membranes to a final concentration of 40 mg/mL. SMA was prepared as 10% (*w/v*) stock using the same buffer. Then, 10% SMA was added to dilute the cell membrane suspension to the concentration of 30 mg/mL and SMA 2.5%. Then, the suspension was rotated at 20 rpm at 4 °C for 7 h to form the native nanodiscs. The insoluble fraction was removed by centrifugation at 100,000× *g* at 4 °C for 1 h. The unbound SMA monomer in the native nanodisc solution was removed by an Amicon Ultra protein concentrator (UFC910024 15ml 30K, Millipore, Merck KGaA, Darmstadt, Germany). Finally, the solution was loaded onto an Ni^2+^ affinity column pre-equilibrated with the equilibrium solution containing 10 mM Tris (pH 7.3), 100 mM NaCl and 2 mM TCEP. The native nanodisc was eluted with the equilibrium solution with an additional 0, 30 or 300 mM imidazole, respectively. The fractions eluted from 30 and 300 mM imidazole were collected for further purification using superose 6 increase 16/300 GL size exclusion chromatography (GE Healthcare, Boston, MA, USA).

### 2.5. Circular Dichroism (CD) 

The liposome sample was prepared by adding 0.5 μL concentrated liposome sample (app. 50 mg/mL) to 125 μL of 20 mM phosphate buffer to a final concentration ~0.2 mg/mL. The native nanodisc sample was prepared by exchanging the buffer to 20 mM phosphate using the protein concentrator. CD measurement was done on the instrument (Chirascan-Plus, Applied Photophysics, Leatherhead, Surrey, UK). The light path was 0.5 mm. The scan speed was 60 nm/min. The temperature was set at 25 °C. The spectrum was collected from 180 to 260 nm and the result was displayed from 190 to 240 nm. The secondary structural analysis was carried out using CDNN software [19].

### 2.6. Transmission Electron Microscopy (TEM)

The TEM sample was prepared by adding a 5 μL sample to a 300-mesh carbon-coated grid (Beijing Zhongjingkeyi Technology, Beijing, China). The liquid was kept for 45 s on the grid before the residual sample was removed by blotting. The grid was then washed using 5 μL ddH_2_O for 45 s. A total of 5 μL 2% uranyl acetate was applied to the grid and removed immediately. After that, another 5 μL 2% uranyl acetate was applied to the grid and kept for 45 s before it was blotted away. The TEM image was acquired at 120 kV (Talos L120C, FEI, Hillsboro, OR, USA). ImageJ (NIH) was used to obtain the size of the particles in the images.

### 2.7. Protein Crosslinking in DM and in Liposomes

Different concentrations of purified SatP in HEPES (pH 7.3), 100 mM NaAc and 4 mM DM were mixed with glutaraldehyde to reach 100 μL with the final concentration of SatP from 0.1 to 0.5 mg/mL and 0.1% for glutaraldehyde. The mixture was placed on ice for various periods from 5 to 30 min until the reaction was ceased with approximately 6.3 μL 1M Tris-HCl pH = 8.6 (the final concentration was 1%, ten-fold the concentration of glutaraldehyde) for the 100 μL reaction system. The SatP liposome sample was diluted to the protein concentration of 10 μM using 20 mM HEPES (pH = 7.3) buffer. A total of 990 μL of the liposome solution was mixed with 10 μL 50 mM BS3 (bis[sulfosuccinimidyl] suberate) (thermo) in DMSO or 10 μL DMSO (as control). The mixtures were allowed to crosslink at room temperature for 1 h. The reaction was then terminated with 20 mM NH_4_HCO_3_. The samples were denatured in a 2×sample loading buffer for SDS-PAGE with excessive SDS (the final concentration is 7.5%). A 4–20% gradient gel (GeneScript, Nanjing, China) was used for the electrophoresis. 

### 2.8. Chemical Shift Prediction

SHIFTX2 (http://www.shiftx2.ca/, accessed on 17 June 2020) was used to predict both the backbone and side chain ^1^H, ^13^C and ^15^N chemical shifts of SatP with its crystal structural coordinates as input (PDB:5YS3). We used the online server to acquire the predicted chemical shifts [20].

### 2.9. Solid-State NMR Experiments

For SSNMR experiments using 3.2 mm rotors, both liposome samples and native nanodiscs were freeze-dried first, and then the dry samples were put into the rotor. The 20% ddH_2_O was added to the dry liposome (~37 mg) and 12 μL ddH_2_O was added to the dry native nanodisc sample (only about 10 mg) to make a full rotor. For the ^1^H-detected experiment, a 5.2 mg dry liposome sample was mixed with a 5.2 μL buffer (10 mM HEPES, 100 mM NaAc, 5% DSS, pH 7.3) first. The sample was then spun down to the rotor using the bruker 0.7 mm centrifugation filling tool with a speed of 5000 rpm for 15 min at room temperature. Only 0.59 μL of sample could be accommodated in a 0.7 mm rotor, and approximately half of the volume was the liposomes and half was the buffer.

All SSNMR experiments were carried out on a 16.45 T (700 MHz ^1^H frequency) Bruker AVANCE NEO spectrometer. A 3.2 mm triple-resonance HCN MAS probe was used to record 1D or 2D ^13^C-detected spectra with the MAS speed at 15 kHz, while a 1.9 mm HCND MAS probe was used to record 1D ^15^N-detected spectra with the MAS speed at 15 kHz. ^1^H-detected spectra were acquired at 100 kHz MAS speed using a 0.7 mm HCN ultrafast MAS probe. The temperature was set to 263 K unless otherwise stated, controlled by a Bruker cooling unit. A temperature calibration was performed using the water peak [21] in the liposome sample for the 3.2 mm probe spinning at 15 kHz, showing it was 286 K. According to the calibration, the setting temperature of 293 K was actually 293 K and the setting temperature of 253 K was 277 K. The temperature calibration was not done for other probes. ^13^C chemical shifts were referenced externally using the DSS scale by calibrating the downfield ^13^C signal of adamantane to 40.48 ppm. The nitrogen dimensions were referenced externally to liquid ammonia (0.00 ppm for NH_3_). The typical recycle delay used for all experiments was 2 s. The NMR data were collected and processed using Topspin (Bruker) and further analysis was carried out using NMRFAM-Sparky [22].

For ^13^C cross-polarization (CP) match using 3.2 mm rotors, the ^13^C rf strength was set to 52 kHz. The CP contact time was 1.2 ms. For ^15^N CP match, the ^15^N rf strength was set to 55 kHz. The contact time was 1.4 ms. ^1^H rf strength was set to *n* = 1 condition with a linear ramp up from 70% to 100%. NCA band-selective transfers were implemented with a 4.5 ms contact time. The ^15^N (centered at 120 ppm) rf spin-lock strength was optimized to 5/2 ωr, and ^13^Ca (centered at 55 ppm) rf spin-lock strength was optimized to near 3/2 ωr. For NCO band-selective transfer, 6.0 ms contact time was used. The ^15^N (centered at 120 ppm) rf spin-lock strength was optimized to near 3/2 ωr and ^13^CO (centered at 174.6 ppm) was optimized to near 5/2 ωr. ^1^H SPINAL-64 decoupling [23] at 83.3 kHz was applied during the evolution and acquisition periods. Dipolar-assisted rotational resonance (DARR) [24,25] mixing scheme with 50 ms was applied for ^13^C-^13^C transfer. States-TPPI [26] sampling mode was used for indirect dimension with the maximum t1 time 8 ms for ^13^C-^13^C and 15 ms for ^15^N-^13^C. 

For the ^1^H detected experiment, all CP transfers were achieved by fulfilling the double-quantum (DQ) transition condition (ω_I_ + ω_S_ = ωr). Linearly ramped-up and ramped-down shapes (10%) with an rf field strength of 80 kHz were applied on ^1^H for all initial and final CP steps with 20 kHz constant pulses on ^15^N or ^13^C, respectively. MISSISSIPPI [27] pulse sequence was used to suppress the water signal with 15 kHz irradiation for 150 ms. WALTZ-16 [28] decoupling (rf field strength 10 kHz) was applied on both ^13^C and ^15^N channels during acquisition. The 2D ^1^H-^15^N spectrum was acquired with an NH CP contact time of 0.6 ms. The 3D hCaNH spectrum was acquired with a 1 ms contact time for hCa and 0.45 ms for NH. The specific CP [29] from Ca (centered at 56 ppm) to N (centered at 120 ppm) was established with ~52 kHz irradiation on ^13^Ca and ~48 kHz irradiation on ^15^N for 8 ms. 

## 3. Results

### 3.1. SatP Forms Hexamers in Native Membranes

**Figure 2:**
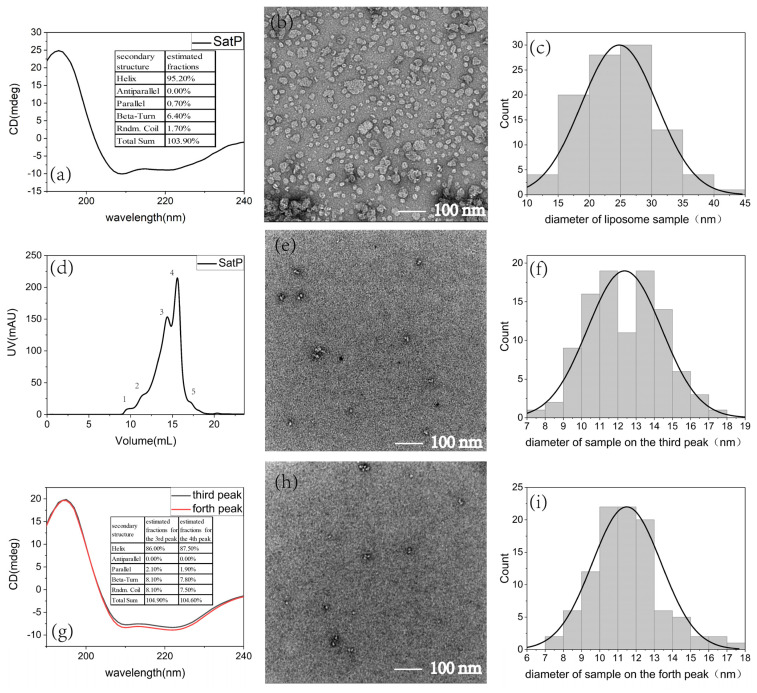
Characterization of the liposome sample and the native nanodisc sample of SatP. The CD spectra of SatP in liposomes (**a**) and in native nanodiscs (**g**) indicated SatP adopting mostly the α-helix structure. (**d**) The purification of native nanodiscs using superose 6 size exclusion chromatography, showing five peaks, the void (1st peak), the 2nd peak, the 3rd peak, the 4th peak and the 5th peak. The 3rd and 4th peaks were what we used for CD and NMR experiments. TEM images of liposomes (**b**) and the native nanodiscs from the 3rd peak (**e**) and the 4th peak (**h**) of size exclusion chromatogram were also shown. The statistical analysis of the particle sizes was shown in (**c**,**f**,**i**) for the liposomes (**c**) and the native nanodiscs (**f**,**i**). (**f**) was for the 3rd peak using 12 TEM images and (**i**) was for the 4th peak using 16 TEM images.

Uniformly ^13^C, ^15^N labeled SatP was expressed and inserted in the membrane in *E. Coli*. For liposome preparation, the protein extract from cell lysis was solubilized in 2% (*w/v*) DM (40 mM) and purified using a nickel affinity column to remove the cell membrane lipids and other protein impurities, as shown in Figure 1b. SatP tends to exhibit different bands on the SDS-PAGE, indicating different oligomeric states. The elution using different concentrations of imidazole all exhibited a dominant band around 75 kDa. Using 300 mM imidazole to elute, the solution showed the minimum molecular weight band around 20 kDa on the SDS-PAGE gel, indicating the monomer band. In between there was a weak band close to 45 kDa and possible other bands. The apparent molecular weights of the bands for SatP oligomers are less than their actual molecular weights, making it difficult to assign the correct oligomeric state for the major band around 75 kDa. To confirm the protein oligomeic state, the protein crosslinking by glutaraldehyde was carried out using purified SatP in DM (Figure 1c and Figure S1a). Both coomasie brilliant blue stains (Figure S1a) and western blot (Figure 1c) were performed to analyze the crosslink results. The control sample of 0.1 and 0.3 mg/mL SatP showed two bands clearly at ~20 and ~75 kDa, respectively. SatP crosslinking for 5 min caused the intensity of the monomer band to decrease for both the protein concentrations (lane 2 compared to lane 1; lane 4 compared to lane 3) as shown in Figure S1a,c. Lane 5 was the 30 min crosslinking product for 0.5 mg/mL SatP, also shown in Figure S1a,c. Increasing the concentration of protein or the crosslinking reaction time would cause more bands to show up, and bands at positions higher than 75 kDa also started to develop. Since there were total six bands from ~ 20 to ~75 kDa, we tentatively assigned these bands, corresponding to oligomers from the monomer to the hexamer. One band at >120 kDa was assigned as the docecamer. Samples from 30 and 300 mM imidazole elution were collected for further liposome preparation. Around 6 mg SatP could be purified from 1 L M9 medium. The CD spectrum of SatP in the liposome indicated a dominant α-helix conformation (Figure 2a).

In order to further confirm the oligomeric state of SatP in the liposome environment, the crosslinking reaction of protein using BS3 was carried out. The results are shown in Figure S1b, indicating both the control SatP liposome sample and the crosslinking product exhibited similar bands. Both showed the darkest band at ~75 kDa as the dominant species (the hexamer band). The crosslinking product also indicated darker bands at the pentamer and tetramer positions compared to the control. Since the crosslinking reaction was carried out in the liposome, the result confirmed the preference of hexameric states for SatP in the liposome.

For native nanodisc preparation, the protein extract from cell lysis was resuspended using 10% (*w/v*) SMA to enclose the protein with its in-situ membrane environment. The nanodisc sample was also purified using a nickel affinity column, as shown in Figure S1c. The 75 kDa band corresponding to the hexameric SatP was clearly seen. The existence of SMA in the loading solution caused the extra smearing image of the SDS-PAGE gel at the lower molecular weight position. Western blot was carried out using the purified SatP sample in SMA after the nickel affinity column, confirming its identity (Figure 1d). The results indicated the oligomeric SatP with the apparent 75 kDa molecular weight was more dominant in this in-situ membrane environment than in the liposomes. A further purification step using superose 6 increase 16/300 GL (GE Healthcare) was only done for native nanodisc samples to obtain a homogeneous size of the nanodisc (Figure 2d). The CD spectra of SatP in native nanodisc also showed a dominant α-helix conformation for the major peaks (3rd and 4th peak) from the size-exclusion chromatography (Figure 2g). 

TEM images of liposomes and native nanodiscs were shown in Figure 2. The diameter of a liposome was averaged to about 25 nm (Figure 2b,c) excluding the rare big structures with the size close to or more than 100 nm. However, the size of a native nanodisc for the 3rd and 4th peak of size-exclusion chromatogram was smaller, between 10–12 nm on average (Figure 2e,f,h,i). The size of native nanodiscs was similar to the literature reports by other laboratories [9,10]. The diameter of the hexameric SatP was around 85 Å according to the crystal structure [11], suggesting mostly only one hexamer would be accommodated in each nanodisc.

### 3.2. Solid-State NMR Reveals Consistent but Slightly Different Structures of SatP in Liposomes and Native Nanodiscs 

**Figure 3:**
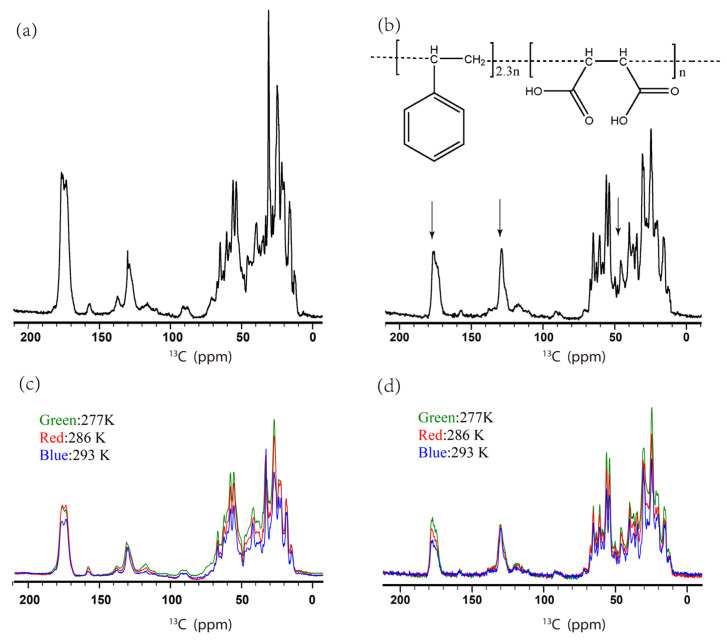
The 1D ^13^C CP spectra of SatP liposomes (**a**) and native nanodiscs (**b**) at 286 K. The SMA chemical structure was also shown in (**b**) with the ratio of S:MA = 2.3:1. Compared to the liposome sample, the native nanodisc sample has 8 times the number of scans. The arrow indicated ^13^C chemical shift positions (~180, ~130 and ~45 ppm) where SMA has some contributions to the peak intensity. The CP spectra were also taken at different temperatures, where the spectra of the liposome sample were shown in (**c**) and the native nanodisc sample in (**d**).

SatP has six transmembrane helices. The large size has made it difficult to reconstitute the protein for a solid-state NMR study. Solid-state NMR studies were carried out on SatP in liposomes and native nanodiscs. The one-dimensional (1D) ^13^C spectra were shown in Figure 3 with the similar resolutions. The natural abundance of ^13^C SMA signals would contribute to the peak intensity around 45, 130 and 180 ppm for a SatP native nanodisc (Figure 3b). The ^13^C signal of SatP native nanodiscs was weaker compared to the liposome sample, because it could only accommodate around 22 mg of hydrated native nanodiscs (4 mg SatP +12 μL ddH_2_O + 2 mg lipids + 4 mg SMA) for a full 3.2 mm rotor while 44 mg of the liposomes (19 mg SatP + 7 μL ddH_2_O + 18 mg lipids) were used to fill up a same size rotor. The temperature effects on the intensity of SSNMR cross-polarization spectra of both samples were also compared, as shown in Figure 3c,d. Both samples showed a continuous decrease in the intensity of ^13^C CP spectra when the sample temperature was increased from 277 to 293 K, indicating motions with the intermediate time scales affecting CP efficiency. 

**Figure 4:**
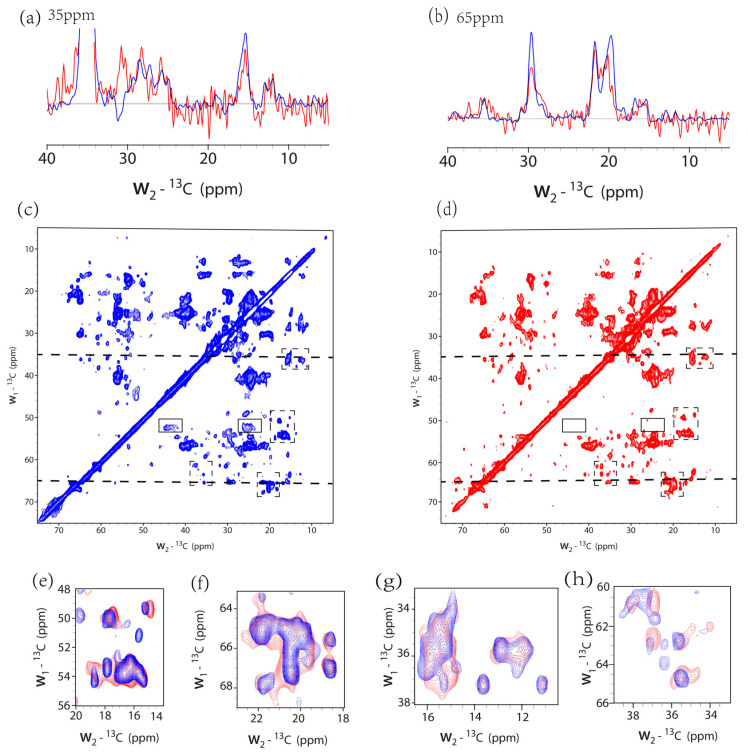
^13^C-^13^C 2D spectra of the SatP liposome sample (protein/lipid mole ratio = 1/28) with 64 scans (blue) and native nanodisc sample with 80 scans (red). The maximum t1 in the indirect dimension was 8 and 7.2 ms, respectively. Squared sine function was used for both spectra, with SSB = 4 for the liposome sample and SSB = 2.5 for the native nanodiscs. The total experiment time was about 27 h for the liposome sample and 24 h for the native nanodisc. The 1D slices at position 35 ppm (**a**) and 65 ppm (**b**) were also displayed for comparison. The linewidth for the peak at 29.5 ppm from the 1D slice of 65 ppm for the liposome sample is around 0.7 ppm. The overlay of the two spectra at four different areas was shown in (**e**–**h**), marked using the rectangles in dashed line in (**c**,**d**).

The 2D ^13^C-^13^C correlation spectra were shown in Figure 4 using 50 ms DARR mixing and an overlay of spectra was shown in Supplement Figure S2. For clarity, only smaller areas were expanded in Figure 4 to show that both spectra exhibited similar peak positions but chemical shift perturbations were also observed for some peaks. It indicated slightly different secondary structures for the protein in the different lipid environments. Interestingly, peaks around (w1 52 ppm, w2 24/44 ppm) were only observed for the liposome samples, indicated in Figure 4c,d. Those were probably from leucine residues in a non-helical conformation. According to the crystal structure, there are seven leucine residues located at regions connecting two transmembrane helices or at the N-, C-terminus, out of a total of 32 leucine residues in the full-length sequence. This difference may suggest different structures or more possibly different dynamics for the protein in the different lipids environments. The 1D slices at 65 and 35 ppm were also displayed for comparison, indicating the weaker intensities for the native nanodiscs. The peaks at 65 and 35 ppm slices correspond to the isoleucine and valine residue peaks in SatP. Our results indicated the preparation of the native nanodiscs was successful. Considering the flat shape of the native nanodisc, it may present a better environment, not distorting the protein structure. 

### 3.3. Solid-State NMR Spectra Assignment of SatP in Liposomes

**Figure 5:**
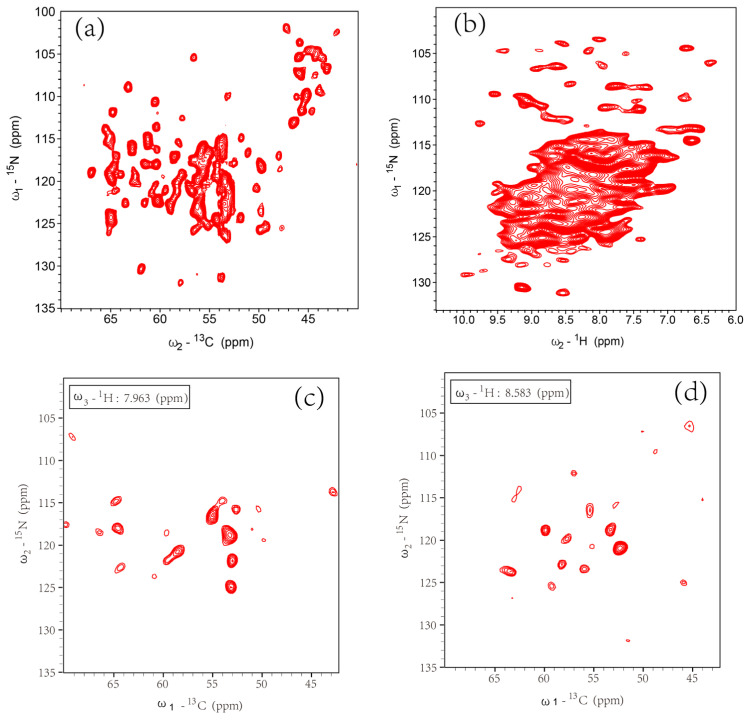
Selected 2D and 3D spectra of the SatP liposome sample. (**a**) NCA spectrum of SatP liposome with 128 total scans and 15 ms the maximum t1 in the indirect dimension. The experiment time was 8 h. (**b**) The 2D ^1^H-^15^N spectrum of the SatP liposome at the MAS speed of 100 kHz with 256 total scans and 22.5 ms the maximum t1 in the indirect dimension. The experiment time was 20 h. The 2D slices of 3D hCaNH spectra at the ^1^H chemical shift of 7.963 ppm (**c**) and 8.583 ppm (**d**). The 3D hCaNH experiment has 128 total scans, and the number of increments for indirect ^13^C dimension was set to 48 with the maximum time 4.8 ms, the number of increments for indirect ^15^N dimension was set to 32 with the maximum time 5.6 ms. The total experiment time was 120 h. (**a**,**b**) were processed using a squared sine function with SSB=3 for both dimensions. hCaNH was processed using EM with LB =0.3 Hz for both F1, F2 and 10 Hz for F3 dimension.

The linewidth for the liposome sample at the 65 ppm slice is 0.7 ppm for the well-separated peak at around 29.5 ppm. The good resolution has prompted us to further obtain ^15^N-^13^C and ^1^H-^15^N heteronuclear correlation spectra. The NCA spectrum displayed good resolutions (Figure 5a), the ^1^H-detected ^1^H-^15^N spectrum showed well-separated peaks only at the glycine region (Figure 5b). However, the number of peaks in the NCA spectrum was less than 100, far less than the number of residues in the protein (188). This observation also suggests the intermediate dynamics existing, causing some residue signal broadening beyond observation. The ^1^H-detected 3 dimensional (3D) hCaNH experiment was also done (Figure 5c,d). However, further 3D spectra were not obtained because the additional signal transfer and mixing caused the signal loss and longer experimental time. The above liposome sample used a protein/lipid (P/L) mole ratio of about 1/28. Adding more protein to the liposome (P/L ratio = 1/15) caused a deterioration of the spectrum resolution (Supplementary Figure S3). Therefore, the assignment of the spectra was only attempted using 2D spectra (Supplementary Figure S4) at P/L ratio = 1/28. 

**Figure 6:**
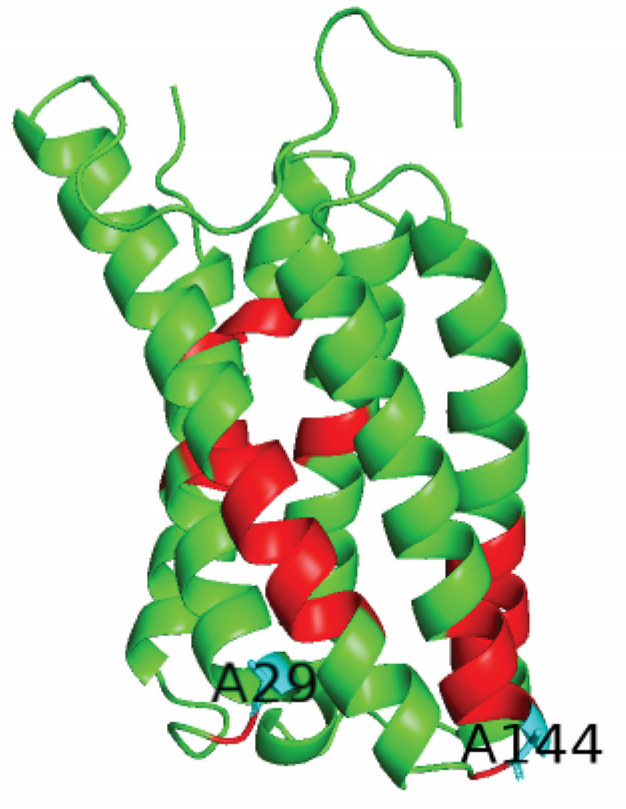
The monomer structure of SatP shows all the assigned residues in red and cyan, where A29 and A144 (in cyan) located at the end of helices are two residues with unique ^13^C chemical shifts.

Utilizing the good resolution of 2D ^15^N-^13^C spectra at glycine ^15^N region, many peak assignments could be achieved. In particular, we assigned one tyrosine-glycine (Y72G73), isoleucine-glycine (I156G157), and cysteine-glycine (C160G161) pair unambiguously since they are unique in the sequence. I184G185 is the second IG pair in the sequence, but it is located outside the helical region close to the C-terminal tail, therefore would have very different ^13^C chemical shifts. We also located all three leucine-glycine pairs (L12G13, L99G100, L103G104), all four alanine-glycine pairs (A29G30, A53G54, A144G145, A153G154), one tyrosine-glycine-glycine triplet (Y45G46G47), one lysine-glycine (K60G61) and two phenylalanine-glycine pairs (F17G18, F140G141). The assignment is shown in Table S1 and the residue positions are indicated in Figure 6. Using the available crystal structure file PDB:5YS3, the ^13^C NMR chemical shifts were predicted using Shiftx2 [20] (http://www.shiftx2.ca/, accessed on 17 June 2020) (Supplementary Figure S4b, Table S1). The ^13^C chemical shifts matches our spectrum well. ^13^C secondary chemical shift values were also obtained using TALOS-N [30]. For all the residues with ^13^C secondary chemical shift values, all but one shows positive values, consistent with the dominant helical structures of the crystal. A144 located at the end of helix 5 has a negative value for the ^13^C secondary chemical shift (~ −0.6); A29 located at the end of helix 1 also has a reduced value of ^13^Cα chemical shifts (51.2 ppm) compared to the A ^13^Cα chemical shifts for the random structure (Cα 52.0 ppm). But the ^13^C secondary chemical shift value for A29 is still positive.

## 4. Discussion

### 4.1. Considerations in the Liposome Sample Preparation

Each SatP molecule has 188 residues, plus the thrombin cleavage sequence (LVPRGS) and the histine tags. In our hands, the highest P/L ratio is 1/15 for liposome preparation. Supposing that all SatP molecules in the liposome are in the hexameric form, the hexamer/lipid ratio is 1/90. Using the diameter of the hexamer (85 Å) and the lipid (~9 Å) [31], we conclude that about 64 lipids are required to surround a SatP hexamer with one circle of bilayer. Thus, 90 lipids would not be enough for two circles. Therefore, it may be too crowded for the protein on the liposome. The reduction of the P/L ratio to 1/28 (the hexamer/lipid ratio 1/168) with an estimated two–three circles of lipids surrounding the hexamer provided SSNMR spectra with better resolutions. However, it also reduced the signal intensity. ^1^H detected SSNMR may provide an approach with a better sensitivity, as indicated by our ^1^H-^15^N HSQC and hCaNH experiments. The size of the liposome may also be an important issue to control. In this research, the liposome was prepared using biobeads to remove the detergent and the size of the liposome was not controlled. The smaller liposome would impose a high curvature on the bilayers and membrane protein, which may be a restriction factor on the spectra resolution.

### 4.2. Considerations in the Native Nanodisc Preparation

Although the ^13^C spectra from native nanodiscs prove the feasibility of retaining the membrane protein native membrane for structural studies, it still faces many obstacles to be a method of choice in SSNMR research. The biggest problem is also the sensitivity. The amount of SatP in native nanodiscs was only about 4 mg in our sample, much less than the liposome sample (~19 mg). In order to pack as much protein as we could to the rotor, lyophilization of the sample was used to remove the extra water. Additional but much less water was added later to the rotor directly to maintain the hydrated membrane environment. The quality of spectra was not affected by this drying and rehydration process as indicated by the linewidth of our spectra. However, native nanodiscs cannot be packed as dense as the liposome once dried, due to the properties of SMA as a rigid organic polymer. Therefore, the amount of protein was significantly reduced in the rotor. The sedimentation method was often used in packing the membrane proteins to a rotor [32], which would be better to be applied to the native nanodisc sample to avoid the sample drying. Furthermore, it would be ideal to study the native membrane without SMA in this sense [7,33]. The study on the bacterial aquaporin Z in its native *E. Coli* membrane was carried out without SMA [7]. An ^19^F NMR study of protein–protein interactions between two mitogen activated protein kinases was also successful using the crude bacterial lysates [33]. However, SMA solubilizing provides us a way to further purify and concentrate the protein. If the expression level of the membrane protein is very high, it may not be necessary to carry out the additional purification. A rough comparison of the ^13^C peak intensity at the 28–32 ppm region where SMA would have no contribution indicated the P/L ratio is about 1/14 for the native nanodisc sample. According to the calculation for the liposome sample, this P/L ratio only allows 1–2 circles of lipids bilayers surrounding a hexamer, consistent with the small size observed in the TEM.

### 4.3. Considerations in the Difference of SatP in Liposomes and Native Nanodiscs

Hexameric SatP is the dominant form in native nanodiscs according to our SDS-PAGE gel result. However, there could be more diversity in the oligomeric species of SatP in liposomes. We observed the chemical shift perturbations between the samples from the two different preparations. The missing peaks in the native nanodiscs could only be partially explained by the lower signal-to-noise ratio. The native membrane is a more inhomogeneous environment and contains a variety of lipids and proteins. This may affect the protein dynamics and structures. Therefore, studying proteins in native membranes would bring different observations and may help us to understand the protein functions more accurately. The transporting of acetate probably involves residues’ motion at the different time scales, which would be interesting to investigate and compare using these two lipids environments. 

## 5. Conclusions

In summary, in this research we have demonstrated SSNMR can be applied to study SatP, a hexamer with multiple transmembrane domains. We showed the similarity and the difference in the spectra of SatP in the liposomes and the native nanodiscs. Considering the preparation of liposomes is not always straightforward for membrane proteins to maintain their functional state, and the native nanodisc is a very promising and simple method. The native nanodisc would even provide a better lipid environment for protein structural studies. The 2D SSNMR studies and ^1^H detection in combination with fast MAS were carried out on SatP liposome samples. However, only a partial sequential assignment was made due to the lack of full sets of 3D SSNMR spectra. The preparation of the SatP liposome and native nanodisc was also described, indicating the intrinsic low sensitivity for membrane proteins with such a size in the SSNMR study. This information would be useful for researchers intending to study membrane proteins with similar sizes. In our opinion, uniformly isotopic labeling in combination with site-selective labeling at strategic locations would be a way to overcome the assignment problem to study the dynamic changes in the membrane protein while the protein is in its functional state in its native membrane.

## Data Availability

The data presented in this study are available on request from the corresponding author.

## Supplementary Materials

The following are available online at https://www.mdpi.com/article/10.3390/life11090908/s1, Figure S1: SDS-PAGE gel of SatP in DM, liposomes and native nanodisc, Figure S2: ^13^C-^13^C 2D spectra of SatP in liposomes and native nanodiscs, Figure S3: ^13^C spectra of SatP liposome with a higher protein concentration (protein/lipid mole ratio=1/15), Figure S4: The 2D spectra of SatP in liposome with assignments, Table S1: The chemical shift assignments of SatP in liposomes based only on 2D NMR correlation spectra.
