# Supplementary material for: Solid-State NMR Studies of the Succinate-Acetate Permease from Citrobacter Koseri in Liposomes and Native Nanodiscs
Source: Life (Basel). 2021 Aug 31;11(9):908. doi: 10.3390/life11090908 (PMC8471396; doi:10.3390/life11090908)
Supplement: Supplementary file 1 [file life-11-00908-s001.zip › life-1321768-supplementary.pdf]

life

MDPI

Article

Supplementary Material

Solid-state NMR studies of the Succinate-acetate Permease from Citrobacter Koseri in Liposomes and Native Nanodiscs

Xing-qi Dong [1,2,3], Jing-yu Lin[1,2,3], Peng-fei Wang[1,2,3], Yi Li [1,2,3], Jian Wang[1], Bing Li[1], Jun Liao [1], Jun-xia Lu [1,*]

[1] School of Life Science and Technology, ShanghaiTech University, Shanghai 201210, P. R. China; dongxq@shanghaitech.edu.cn, linjy@shanghaitech.edu.cn, wangpf@shanghaitech.edu.cn, liyi@shanghaitech.edu.cn, wangjian1@shanghaitech.edu.cn, libing@shanghaitech.edu.cn, liaojun@shanghaitech.edu.cn
[2] State Key Laboratory of Molecular Biology, CAS Center for Excellence in Molecular Cell Science, Shanghai Institute of Biochemistry and Cell Biology, Chinese Academy of Sciences, Shanghai 200031, P. R. China
[3] University of Chinese Academy of Sciences, Beijing 100049, P. R. China
* Correspondence: lujx@shanghaitech.edu.cn;

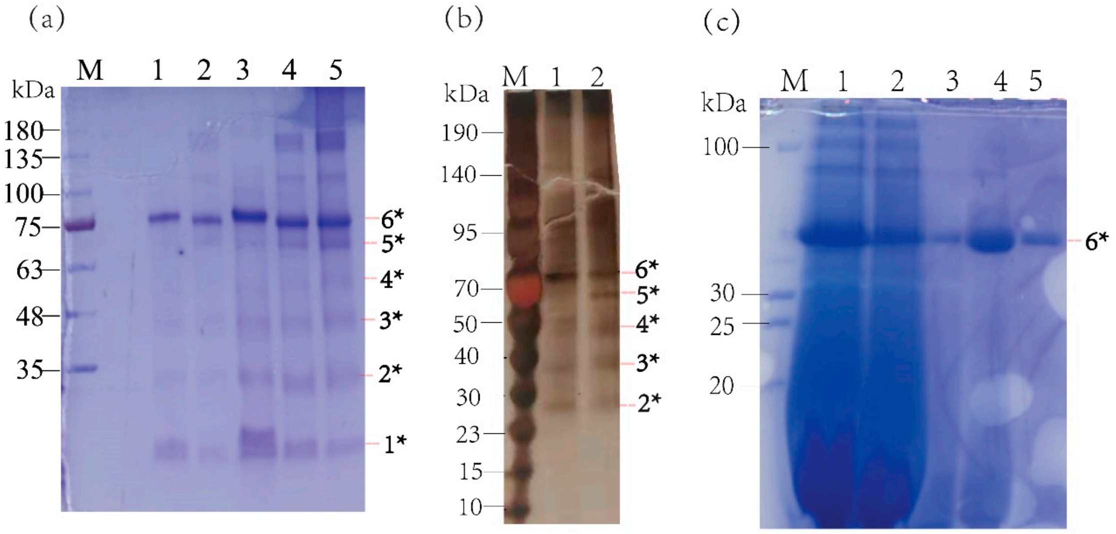

Figure S1. (**a**) SDS-PAGE gel in the coomassie brilliant blue stain showed SatP in different oligomeric state in DM. The protein was crosslinked using glutaraldehyde. lane 1–5 are the same as figure 1(c) described. (**b**) SDS-PAGE gel in silver stain showed the oligomeric states of SatP in liposomes, where lane 1 was the control sample of SatP liposome without crosslinking, lane 2 was the crosslinking product using BS3 (bis[sulfosuccinimidyl] suberate) (Thermo). (**c**) SDS-PAGE gel in the coomassie brilliant blue stain showed the results of native nanodisc preparation. The protein extraction using SMA, lane 1; lane 2-5 are the same as figure 1(b) described. n* indicates different oligomeric state of SatP.

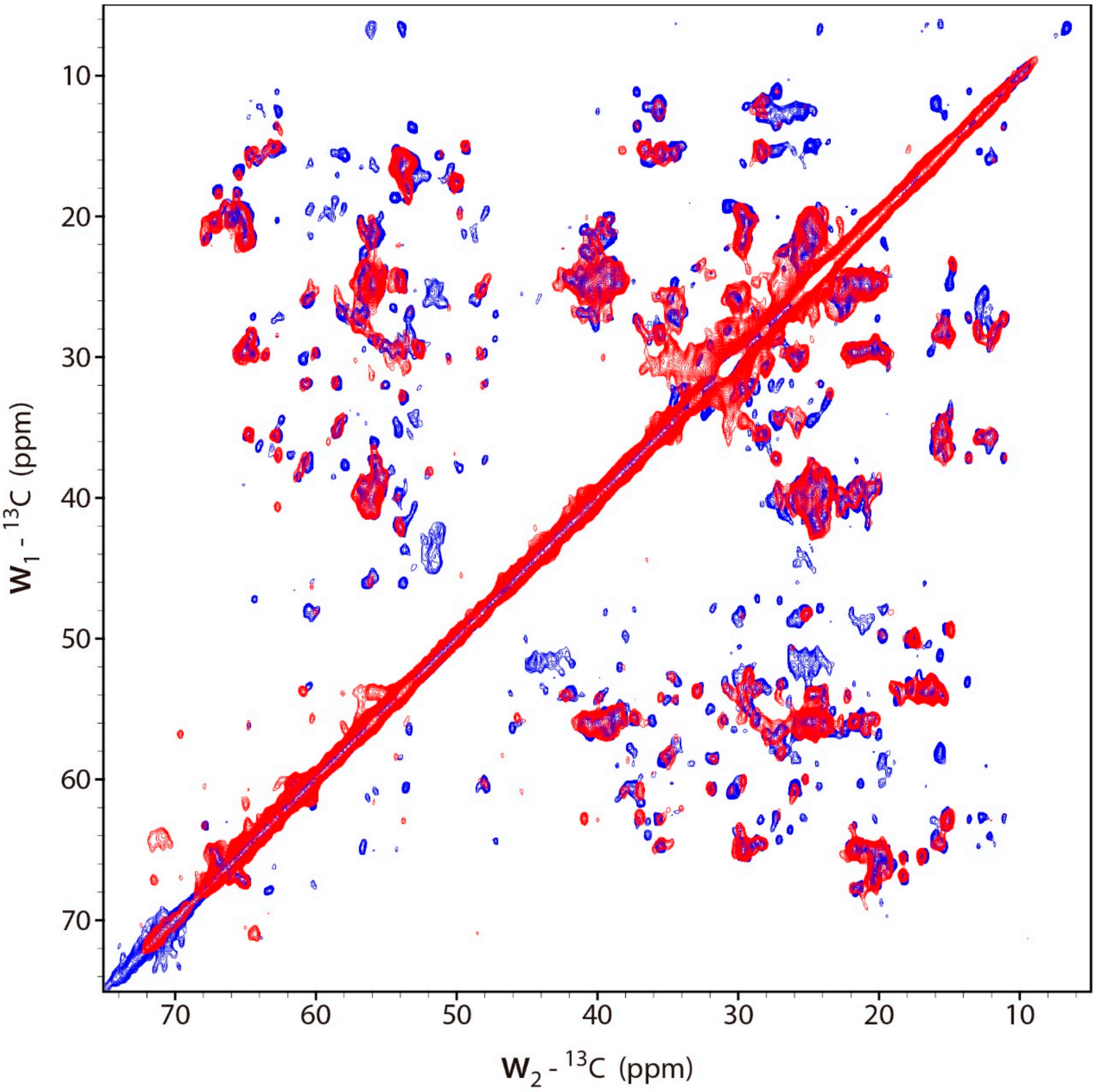

Figure S2. ^{13}C-^{13}C 2D spectra of SatP in liposomes (protein/lipid mole ratio = 1/28) (blue) and in native nanodiscs (red). The maximum t1 in the indirect dimension was 8 ms and 7.2 ms, respectively. Squared sine function was used for both spectra, with SSB = 4 for the liposome sample and SSB = 2.5 for the native nanodiscs. The total experiment time was about 27 h for the liposome sample and 24 h for the native nanodisc.

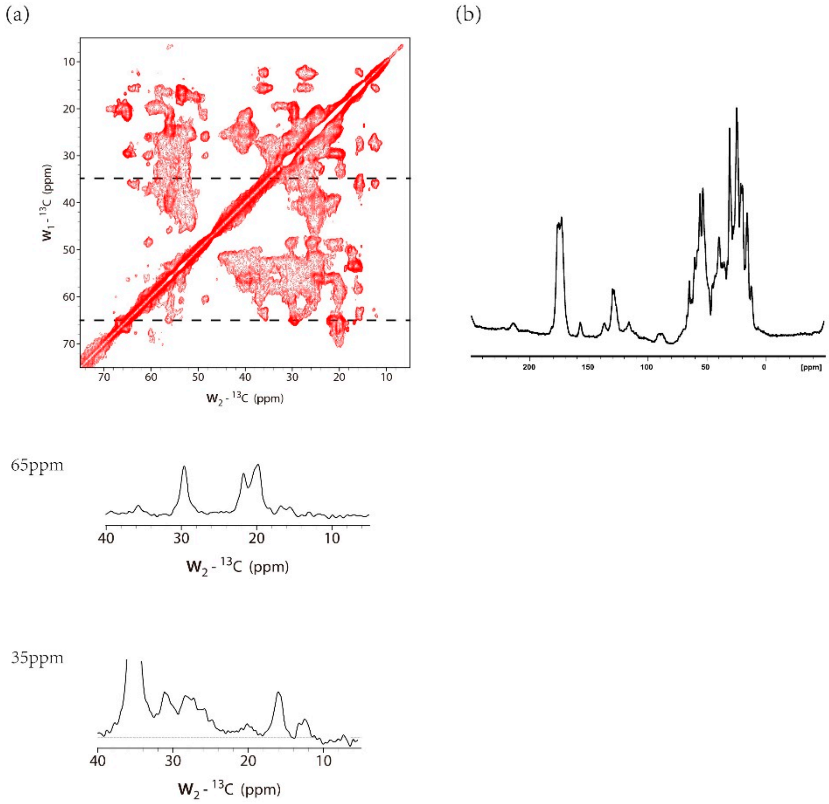

Figure S3. ^{13}C spectra of SatP liposome with a higher protein concentration (protein/lipid mole ratio = 1/15). (**a**) ^{13}C-^{13}C 2D spectrum with 1D slices at position 65 ppm and 35 ppm. The linewidth for the peak at 29.5 ppm from the 1D slice of 65 ppm is around 1 ppm. (**b**) 1D ^{13}C CP spectrum of SatP liposome.

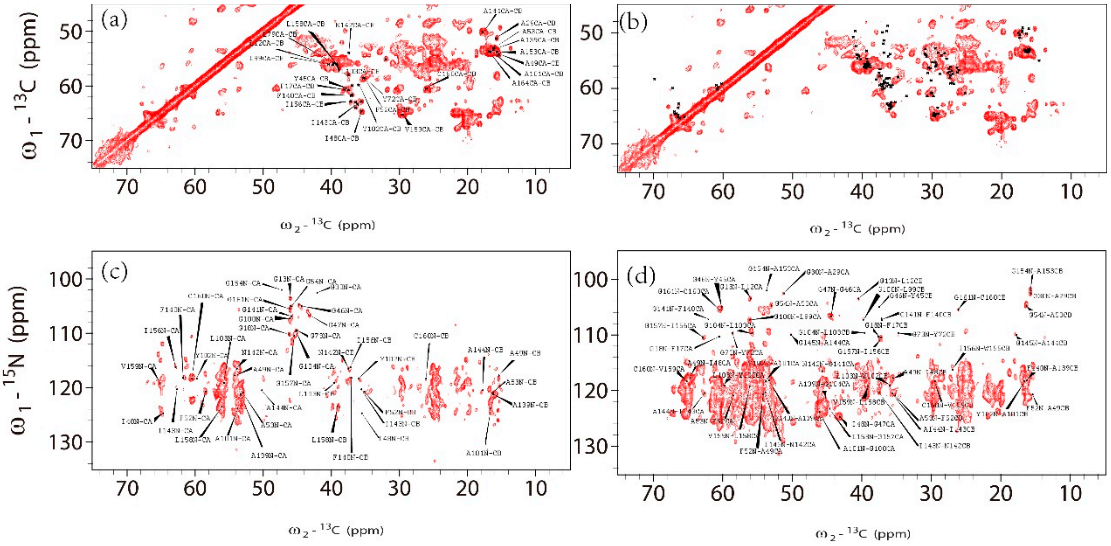

Figure S4. The 2D spectra of SatP in liposome with some assignments labeled. ^{13}C-^{13}C 2D spectrum (**a**); NCACX (**c**) and NCOCX (**d**) spectrum. NCACX experiment was acquired with 256 scans and 112 t1 increments (maximum t1 15 ms). The total experiment time was 16 h. NCOCX experiment was acquired with 768 scans and 112 t1 increments (maximum t1 15 ms). The total experiment time was 48 h. The ^{13}C chemical shift prediction (**b**) using Shiftx2 (http://www.shiftx2.ca/) based on the crystal structure (pdb: 5ys3) was also indicated on ^{13}C-^{13}C 2D spectrum.

Table S1. The chemical shift assignments of SatP in liposomes based only on 2D NMR correlation spectra.

	**Experiment Results**				Shiftx2 Predication		Secondary Chemical Shift	2nd Structure
	**aa**	**^{15}N**	**^{13}Cα**	**^{13}Cβ**	^{13}Cα	^{13}Cβ		
1	L12		56.0	40.5	56.8	39.9	2.7	
2	G13	103.5	46.0		45.9			
3	F17		60.5	37.6	60.1	37.5	3.9	Helix 1
4	G18	110.3	46.2		46.0			
5	A29		51.2	15.7	50.6	16.9	2.5	
6	G30	102.5	42.1		43.7			
7	Y45		60.2	37.5	58.2	37.9	3.1	
8	G46	105	44.4		45.5			
9	G47	106.7	43.2		44.1			
10	I48	124.7	64.7	35.5	63.4	36.6	5.9	Helix 2
11	A49	117.8	54.2	15.4	53.8	16.7	5.8	
12	F52	120.7	58.5	35.2	58.6	36.8	4.3	
13	A53	120.9	53.0	16.3	53.5	16.4	3.7	
14	G54	104.7	45.8		46.1			
15	Y72		58.6	34.7	58.3	35.3	4.3	Helix 3
16	G73	109.6	45.2		45.8			
17	L99		56.1	39.8	56.5	39.9	3.5	
18	G100	107.3	46.1		46.1			
19	A101	123.9	53.8	16.6	53.9	16.7	4.2	Helix 4
20	Y102	118.4	59.8	36	59.8	36.1	4.2	
21	L103	119.1	55.8	39.6	56.6	40.0	3.4	
22	G104	109.6	45.2		45.9			
23	A139		53.3	16.3	53.8	16.9	4.0	
24	F140	118.2	61.8	37.1	59.7	37.7	5.7	
25	G141	107.3	45.7		46.2			
26	N142	116.5	53.9	37.5	54.1	37.3	1.8	Helix 5
27	I143	120.3	62.7	35.6	63.2	36.9	3.8	
28	A144	120.5	50.2	17.8	50.0	17.5	-0.6	
29	G145	110	43.9		44.8			
30	A153		53.7	15.7	53.8	16.6	5.0	
31	G154	102	47.3		45.9			
32	W155			26.9	59.5	27.5		
33	I156	116.2	62.9	37.2	62.9	35.7	2.4	
34	G157	110.5	45.5		46.1			Helix 6
35	L158	123.5	56.1	39.1	56.5	39.9	4.2	
36	V159	119.2	65.2	29.5	65.1	30.1	5.2	
37	C160	118.4	60.5	26.1	61.3	25.7	3.1	
38	G161	105.5	46.1		45.9			

The secondary chemical shifts were calculated (ΔδCα-ΔδCβ) for residues with Cβ assignments. The ^{13}C chemical shift obtained from Shiftx2 predication was also shown for the corresponding sites. The respective 2nd structure region of the protein was labeled according to the crystal structure PDB:5YS3.
